# Prevention of Muscle Atrophy by Low-Molecular-Weight Fraction from *Hirsutella sinensis* Mycelium

**DOI:** 10.3390/cimb46120839

**Published:** 2024-12-12

**Authors:** Yi-Wen Chen, Tsung-Ju Li, Li-Ching Wang, Bi-Hua Yang, Yen-Lien Chen, Chin-Chu Chen, Hsin-Tang Lin

**Affiliations:** 1 Biotech Research Institute, Grape King Bio Ltd., Taoyuan City 325, Taiwan; yiwen.chen@grapeking.com.tw (Y.-W.C.); tsungju.li@grapeking.com.tw (T.-J.L.); sybil.yang@grapeking.com.tw (B.-H.Y.); Ian.chen@grapeking.com.tw (Y.-L.C.); 2 Department of Food Safety, National Chung Hsing University, Taichung City 402, Taiwan; gash94272abc@gmail.com; 3 Department of Food Science, Nutrition, and Nutraceutical Biotechnology, Shih Chien University, Taipei City 104, Taiwan; 4 Institute of Food Science and Technology, National Taiwan University, Taipei City 106, Taiwan; 5 Department of Bioscience Technology, Chung Yuan Christian University, Taoyuan City 320, Taiwan

**Keywords:** *Hirsutella sinensis* mycelium, sarcopenia, muscle atrophy, C2C12 cell

## Abstract

Muscle atrophy, an age-related condition, presents a growing healthcare concern within the context of global population aging. While studies have investigated *Hirsutella sinensis* for its potential antifatigue properties, reports on its active components remain limited. This study evaluated the efficacy of *H. sinensis* mycelium extract on muscle health, utilizing a 1:1 water–ethanol preparation administered to C57BL/6 mice exhibiting acute hind leg atrophy. The results indicated no adverse effects, with significant improvements in muscle endurance and soleus muscle mass observed over a 14-day period. To further elucidate the mechanisms and effects of *H. sinensis* mycelium on dexamethasone-induced muscle atrophy, the water extract was fractionated into components of <3.5 kDa, 3.5–10 kDa, and >10 kDa using dialysis membranes. The investigation utilized a C2C12 cell atrophy model, induced by dexamethasone, to analyze the expression of relevant genes via qPCR. The results demonstrated that the <3.5 kDa and >10 kDa fractions significantly upregulated the expression of *Myh2* and *Myh7* genes while simultaneously downregulating the expression of *MuRF-1* and *Atrogin-1*. It is noteworthy that the <3.5 kDa fraction exclusively enhanced MYHC protein expression and suppressed AMPK expression, as confirmed by Western blot analysis. This comprehensive pilot study suggests that the low-molecular-weight fraction of *H. sinensis* mycelium exhibits considerable potential for muscle mass preservation and atrophy mitigation. As a result, it offers a promising direction for the development of supplements aimed at addressing fatigue and preventing muscle atrophy.

## 1. Introduction

Sarcopenia is a complex condition linked to aging that not only reduces the quality of life of elderly people but also affects physical functionality and metabolism, leading to increased risks of diseases and mortality [1]. With the rapid increase in the global elderly population, sarcopenia has emerged as a major public health concern for older adults [2]. During the aging process, the imbalance between protein synthesis and degradation contributes primarily to the progressive loss of muscle function. The ubiquitin–proteasome pathway plays a crucial role in the degradation of skeletal muscle proteins, with the activation of ubiquitin ligases directly leading to a reduction in muscle mass [3]. Among the various E3 ubiquitin ligases, *MuRF-1* and *Atrogin-1* are specific E3 ubiquitin ligase genes. Overexpression of these genes leads to muscle atrophy [4]. *MuRF-1* primarily targets contractile and structural proteins, such as Myosin Heavy Chain, titin, and myosin-binding protein C [5]. *Atrogin-1* regulates muscle synthesis and regeneration, enhances the expression of muscle structural proteins, and promotes skeletal muscle fiber hypertrophy [6,7]. Although aging inevitably leads to a decline in physical function, appropriate therapeutic or preventive interventions can help reduce its adverse effects. Currently, there are no approved pharmacological treatments for the prevention of sarcopenia. Dietary modifications and resistance exercises are the primary strategies for improvement [8].

*Ophiocordyceps sinensis* (*O. sinensis*) is a fungus that parasitizes the larvae of Lepidoptera and has been used in China for thousands of years as a traditional medicine and health food [9]. Studies have shown that *O. sinensis* possesses various bioactivities, including modulating immune responses, inhibiting tumor cell proliferation, enhancing liver function, regulating insulin sensitivity, reducing plasma cholesterol levels, and modulating steroidogenesis [10]. Active compounds identified in *O. sinensis*, such as cordycepin, nucleotides, exopolysaccharides, peptides, and sterols, have been shown to have various pharmacological effects [11]. *Hirsutella sinensis* (*H. sinensis*), an anamorph (asexual stage) of *O. sinensis*, has a long consumption history in Eastern cultures [12]. Rich in bioactive compounds such as cordycepin, adenosine, polysaccharides, vitamins, and trace elements, it is not only considered a traditional Chinese medicine for treating diseases and enhancing body strength but also a tonic for reducing fatigue [13]. Its mycelium has been found to have therapeutic effects on pulmonary inflammation and fibrosis [14], with potential benefits for treating heart failure [15] and chronic bronchitis [16]. Research has demonstrated significant effects of *H. sinensis* mycelium in reducing fatigue and enhancing exercise endurance in vivo [17]. A recent investigation revealed that *Hirsutella sinensis* effectively mitigated pathological changes in gastrocnemius muscles within the SOD1^G93A^ transgenic mouse model [18]. While these findings demonstrate the significant effects of *Hirsutella sinensis* on muscle tissue, the variations in muscle benefits observed across different production methods warrant additional research to elucidate the precise mechanisms of its bioactive constituents.

This study demonstrated the effectiveness of *H. sinensis* mycelium in reducing muscle fatigue and atrophy in a murine model. In parallel, a cellular model was developed using dexamethasone-treated C2C12 cells to simulate muscle atrophy, with different extracted fractions applied as treatments. Gene expression and protein expression were analyzed at both cellular and molecular levels through quantitative real-time PCR (qRT-PCR) and Western blot analyses. The goal was to identify the most potent fraction of *H. sinensis* extract capable of inhibiting muscle atrophy pathways, with the long-term aim of developing a natural supplement to combat chronic muscle loss.

## 2. Materials and Methods

### 2.1. Materials and Reagents

*Hirsutella sinensis* mycelium was provided by Grape King Bio (Taoyuan, Taiwan). Dexamethasone was purchased from Merck Ltd. (Darmstadt, Germany). Fetal Bovine Serum and Horse Serum were obtained from Hyclone (Logan, UT, USA). Anti-AMPKα1/2 was purchased from Santa Cruz in the USA. Anti-Myosin Heavy Chain was obtained from R&D Systems (Minneapolis, MN, USA). Anti-α-Tubulin was purchased from Merck (Darmstadt, Germany). Goat Anti-Mouse IgG HRP Conjugated and Goat Anti-Rabbit IgG HRP Conjugated were purchased from BETHYL (New Taipei City, Taiwan).

### 2.2. Cultivation and Sample Preparation

The mycelium of *H. sinensis* was cultured in a 20-ton fermenter at 18 °C, 30 rpm, with an aeration rate of 500 L/min for nine days. Following fermentation, the samples were concentrated, freeze-dried, and ground into powder to obtain the freeze-dried powder of the *H. sinensis* mycelium fermentation broth. The freeze-dried powder was then extracted using ethanol (HS_EtOH) and water (HS_W) for subsequent in vitro analysis and in vivo study.

### 2.3. Animal Maintenance and Treatment

For the disuse atrophy animal model, 4-week-old male C57BL/6J mice were obtained from the National Laboratory Animal Center (Taipei, Taiwan), with approval from the Institutional Animal Care and Use Committee of the Medical and Pharmaceutical Industry Technology and Development Center, New Taipei City, Taiwan (IACUC-1090006). The mice were housed for an additional 2 weeks in a controlled environment (ambient temperature: 23 ± 1 °C, relative humidity: 60 ± 5%, 12:12 h light/dark cycle) before the experiment. They had ad libitum access to diet and purified water. The mice were then divided into three groups: sham (healthy control), vehicle (control), and *Hirsutella sinensis* mycelium (HS). HS treatments containing a 1:1 ratio of ethanol and water extracts were administered orally via a gastric tube (500 mg/kg). Muscle loss was induced through cast immobilization (IM) for 7 days. Muscle strength (measured using the GSM Grip-Strength Meter, #47200, Ugo Basile) and endurance tests (Rodent Treadmill, #47300, Ugo Basile) were conducted on day 7 and 7 days post-cast removal. After the experiment, the mice were euthanized, and their hind limb skeletal muscles were harvested and weighed. Muscle endurance was assessed by recording the number of times the mice touched an electric shock sensor due to fatigue during the treadmill test.

### 2.4. Cell Culture and Treatment

C2C12 cells (ATCC CRL-1772; Manassas, VA, USA) should be cultured in Dulbecco’s Modified Eagle Medium (DMEM) supplemented with 10% Fetal Bovine Serum (FBS) and 1% Penicillin–Streptomycin (P/S). For differentiation, we use DMEM supplemented with 2% Horse Serum and 25 mM Dexamethasone dissolved in DMSO. Seed C2C12 cells at 6 × 10^4^ cells per 6 cm culture dish and culture in a growth medium at 37 °C with 5% CO_2_. Upon reaching confluence, change to a differentiation medium with 2% Horse Serum. Change the differentiation medium every two days for four days to induce myoblast fusion and myotube differentiation.

### 2.5. Cell Viability

The cell viability was assessed using a commercial MTT test. After removing the old culture medium, the MTT reagent was diluted with PBS, added to each well of a 96-well culture plate, and incubated at 37 °C with 5% CO_2_ for 1 h. After incubation, the supernatant was removed, and DMSO was added to dissolve the formazan crystals. Absorbance was then measured at 570 nm using a microplate reader.

### 2.6. Analysis of mRNA Gene Expression

The mRNA was extracted using the GeneJET RNA Purification Kit (Thermo Fisher Scientific, Lithuania). The concentration and purity were measured using a Nanodrop spectrophotometer (Cambridge, UK). For cDNA synthesis, mRNA was reverse transcribed using the iScript™ cDNA Synthesis Kit (Bio-Rad, Hercules, CA, USA). The resulting cDNA was used for quantitative PCR (qPCR) with the CFX96 Connect Real-Time PCR System (Bio-Rad, USA). A melt curve analysis was performed to generate the dissociation curve.

### 2.7. Western Blot Analysis

After removing the cell supernatant, the cells were washed, and RIPA lysis buffer (Visual Protein, Taiwan) was added to lyse the cells. The lysate was collected and centrifuged to obtain the protein extract, which was stored at −20 °C. Protein quantification was carried out using a BCA Protein Assay Kit (Thermo Fisher Scientific, Lithuania). The protein extracts were then denatured, separated using SDS–polyacrylamide gel electrophoresis, and transferred onto a PVDF membrane. After blocking, the membrane was incubated with primary and secondary antibodies and then developed using a chemiluminescent substrate for imaging and band intensity quantification (Bio-Rad imaging system; ChemiDoc XRS+, USA).

### 2.8. Statistical Analysis

All experiments were performed three times, and the data are presented as the mean ± SD. Statistical analysis was conducted using GraphPad Prism (version 8.0) and included one-way ANOVA followed by Dunnett’s post hoc test. A *p*-value of less than 0.05 (*p* < 0.05) indicated a significant difference between groups.

## 3. Results

### 3.1. Effects of H. sinensis Extract on Body Weight and Food Intake in Immobilization-Induced Sarcopenic Mice

**Figure 1:**
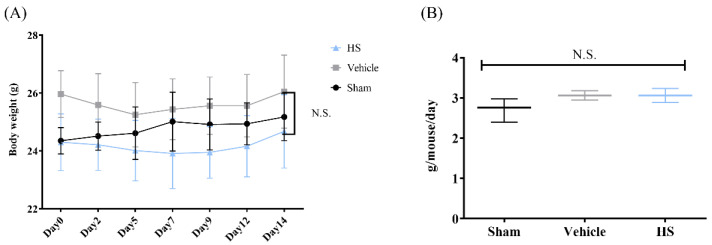
Effect of *H. sinensis* extract powder on IM-induced sarcopenia mice (*n* = 4). (**A**) Body weight; (**B**) food intake. N.S., no statistical significance.

Body weight is an important indicator of the health status of mice and can be used to evaluate any potential side effects of *H. sinensis* extract. Figure 1A shows the changes in body weight over two weeks. Throughout the experiment, we observed no deaths or abnormal clinical or physiological signs, and there were no significant differences in the initial and final body weights among the groups (*p* > 0.05). Figure 1B displays the changes in food intake over the same period. Although there was a tendency for increased food intake in the mice that were immobilized (HS group) compared to the non-immobilized group (sham group), the statistical analysis showed that neither immobilization nor HS feeding significantly affected food intake (*p* > 0.05). Overall, under the experimental conditions, *H. sinensis* treatment did not have a significant impact on the body weight or food intake of the mice, indicating a lack of notable side effects.

### 3.2. Effects of H. sinensis Extract on Hindlimb Grip Strength and Muscle Endurance in Sarcopenic Mice

**Figure 2:**
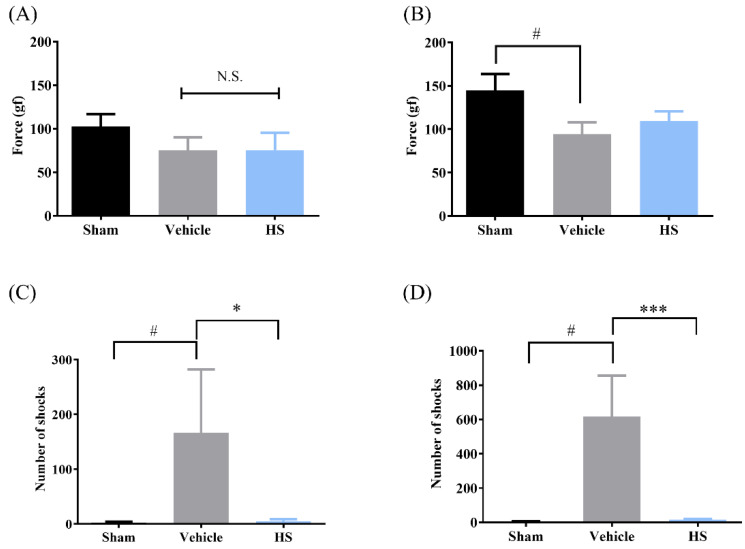
Effects of *H. sinensis* powder administration on grip strength and muscle endurance in mice (*n* = 4). (**A**) Grip strength after 7 days. (**B**) Grip strength after 14 days. (**C**) Muscle endurance after 7 days. (**D**) Muscle endurance after 14 days. Values with * have a significant difference (*p* < 0.05); *** means *p* value is <0.001; # means *p* value < 0.05 compared to sham; N.S., no statistical significance.

Figure 2 illustrates the impact of sarcopenia on hindlimb grip strength and muscle endurance in mice. The results of the grip strength test after 7 days of IM are presented in Figure 2A, showing a significant reduction in hindlimb grip strength. However, there was no significant difference in grip strength between the HS group and the vehicle group (*p* > 0.05). When the observation period was extended to 14 days (Figure 2B), although the grip strength in the HS group did not reach a statistically significant improvement, there was a noticeable trend toward recovery. This suggests that prolonged administration of the extract may contribute to the restoration of grip strength in the context of muscle atrophy. Figure 2C,D show the effects of sarcopenia on muscle endurance in mice. After 7 days of immobilization (Figure 2C), there was a marked decline in muscle endurance. At this stage, the HS group showed a significant improvement in muscle endurance compared to the vehicle group (*p* < 0.05). When the observation period was extended to 14 days (Figure 2D), the sarcopenic mice exhibited a significant increase in the number of electric shocks, indicating a further deterioration in muscle endurance. In contrast, the HS group demonstrated a significant recovery in muscle endurance (*p* < 0.001). In summary, the findings suggest that *H. sinensis* extract significantly enhances muscle endurance in an immobilized model and shows potential for restoring grip strength over a prolonged observation period.

### 3.3. Effects of H. sinensis Extract Powder on Gastrocnemius and Soleus Muscle Mass in Sarcopenic Mice

**Figure 3:**
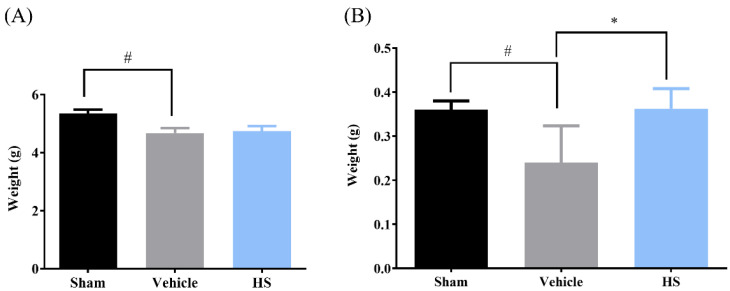
Effect of *H. sinensis* powder feeding for 14 days on the muscle mass of mice (*n* = 4) in the gastrocnemius and soleus muscles. (**A**) Gastrocnemius muscle. (**B**) Soleus muscle. Values with * have a significant difference (*p* < 0.05) compared to vehicle; # means there is a significant difference (*p* < 0.05) compared to sham.

After a two-week trial period, the mice were humanely euthanized, and their gastrocnemius and soleus muscles from the hindlimbs were carefully removed and weighed. Figure 3A shows the effects of *H. sinensis* extract treatment on the gastrocnemius muscle mass in sarcopenic mice. The results indicate that the gastrocnemius muscle mass in the vehicle group significantly decreased compared to the sham group. However, administration of *H. sinensis* extract powder did not result in a significant change in gastrocnemius muscle mass. Figure 3B illustrates the effects on soleus muscle mass. The data also demonstrate a significant reduction in soleus muscle mass in the cast-immobilized group compared to the sham group (*p* < 0.05). However, compared to the vehicle group, the administration of *H. sinensis* extract significantly increased soleus muscle mass (*p* < 0.05). These findings suggest that *H. sinensis* extract has a notable positive effect on soleus muscle mass in immobilization-induced muscle atrophy in mice, indicating its potential anti-sarcopenic effects in specific skeletal muscle groups.

### 3.4. Initial Screening of Effective Fractions by Western Blot

**Figure 4:**
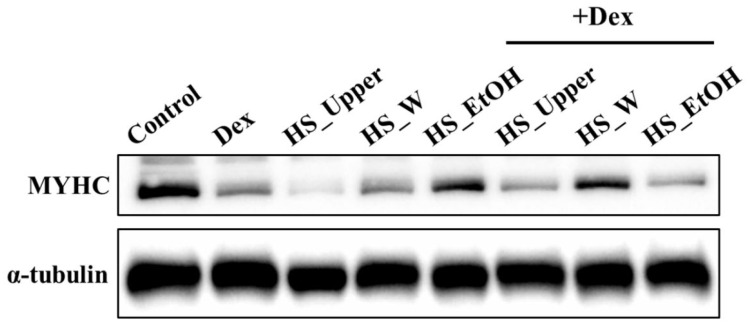
Evaluation of the efficacy of different solvent extracts of *H. sinensis*.

The animal experiments involving mixes of *H. sinensis* in water and ethanol extracts in equal proportions showed potential effects in reducing muscle atrophy. To further identify the active components, we used Western blot analysis to examine different solvent-extracted fractions of *H. sinensis*. After the fermentation process, we separated the fermentation supernatant (HS_Upper) from the mycelium by centrifugation. The mycelium was then extracted using either water or ethanol, resulting in the HS_W (water extract) and HS_EtOH (ethanol extract) samples. These samples were tested on a C2C12 myoblast cell model to evaluate the protein expression of Myosin Heavy Chain (MYHC), a key marker of mature muscle fibers. Figure 4 shows that the *H. sinensis* water extract (HS_W) significantly increased MYHC protein expression (*p* < 0.05) under dexamethasone induction, outperforming the HS_EtOH fraction. This suggests that HS_W has a stronger protective effect against dexamethasone-induced muscle damage. This result suggests that the active components of *H. sinensis* are mainly located in the aqueous extract of the mycelium. Therefore, subsequent experiments will focus on further fractionating the aqueous extract based on molecular weight.

### 3.5. Effects of Different-Molecular-Weight Fractions of H. sinensis Extracts on C2C12 Cell Viability

**Figure 5:**
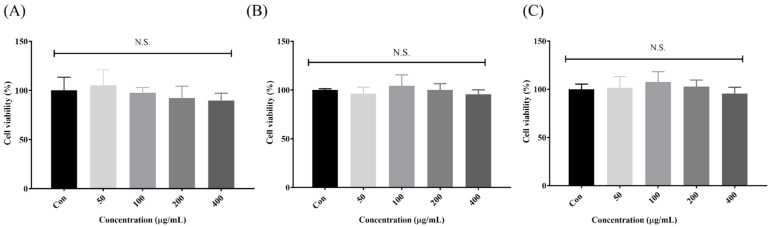
Effects of different-molecular-weight fractions on C2C12 cell viability: (**A**) <3.5 kDa, (**B**) 3.5–10 kDa, and (**C**) >10 kDa. Results are expressed as mean ± SD (*n* = 3). N.S., no statistical significance.

C2C12 mouse myoblast cells, which were differentiated into myotubes, were used in the study to examine how different-molecular-weight fractions of *H. sinensis* extracts affect cell viability. We used the MTT assay to assess the impact of these fractions at various concentrations (50, 100, 200, 400 μg/mL) over 24 h. Figure 5A–C indicate that the cell viability at different concentrations of *H. sinensis* extracts did not significantly differ from the control group (*p* > 0.05), suggesting the absence of cytotoxicity.

### 3.6. Real-Time Quantitative PCR Analysis of Molecular Weight Fractions of H. sinensis Extracts on Gene Expression in C2C12 Cells

Based on previous animal studies, as shown in Figure 3B, feeding *H. sinensis* extract resulted in a significant improvement in the mass of the soleus muscle in cast-immobilized mice. A previous study demonstrated that the gene expression in the soleus muscle is primarily regulated by *Myh2* and *Myh7* genes [19]. Therefore, in this study, different-molecular-weight fractions of *H. sinensis* water extract (100 μg/mL) were added to differentiated C2C12 cells to analysis the fractions’ effect.

**Figure 6:**
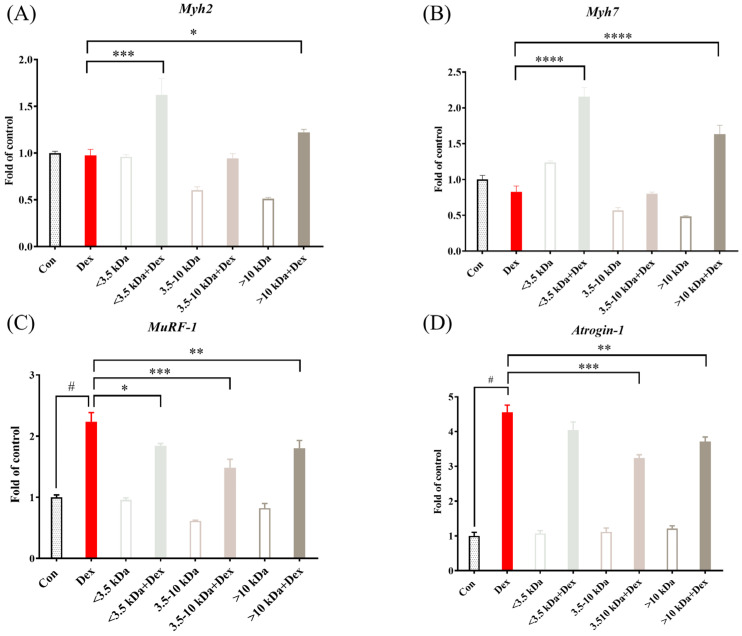
Gene expression levels in sarcopenic cells treated with different-molecular-weight fractions of *H. sinensis* aqueous extract. (**A**) *Myh2;* (**B**) *Myh7;* (**C**) *MuRF-1;* (**D**) *Atrogin-1*. Results are expressed as mean ± SD (*n* = 3). Compared with the dexamethasone group, * *p* < 0.05, ** *p* < 0.01, *** *p* < 0.001, and **** *p* < 0.0001. Compared with the control group, # *p* < 0.05.

As shown in Figure 6A, although there were no significant differences in *Myh2* expression between the dexamethasone-induced group and the control group (*p* > 0.05), the addition of <3.5 kDa and >10 kDa fractions significantly increased *Myh2* gene expression (*p* < 0.05). Figure 6B demonstrates that while *Myh7* gene expression showed a decreasing trend under dexamethasone treatment, the addition of <3.5 kDa and >10 kDa fractions significantly enhanced *Myh7* gene expression (*p* < 0.05). Additionally, Figure 6C shows that dexamethasone significantly increased *MuRF-1* gene expression (*p* < 0.05), indicating increased protein degradation in muscle atrophy. However, the addition of different-molecular-weight fractions significantly inhibited *MuRF-1* gene expression, with the 3.5–10 kDa fraction showing the most pronounced effect. Figure 6D illustrates that dexamethasone significantly elevated *Atrogin-1* gene expression (*p* < 0.05), indicating its role in protein degradation. After the addition of different-molecular-weight fractions, all fractions reduced *Atrogin-1* gene expression, with the 3.5–10 kDa fraction exhibiting the strongest inhibition.

### 3.7. Western Blot Analysis of the Effects of Molecular Weight Fractions of H. sinensis Extract on MYHC and AMPK Expression in C2C12 Cells

**Figure 7:**
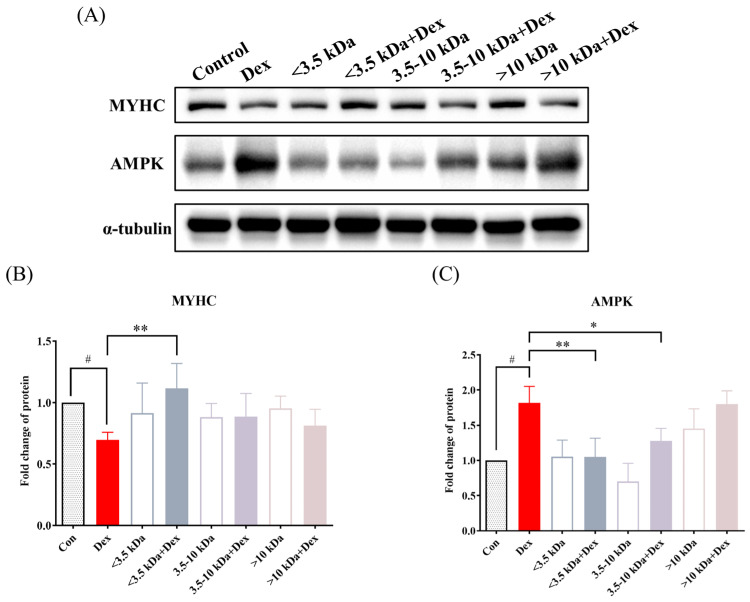
Effects of different-molecular-weight fractions of *H. sinensis* aqueous extract on MYHC and AMPK protein expression in sarcopenic C2C12 cells. (**A**) The results of protein expression in different extract fractions; (**B**) MYHC; (**C**) AMPK. Results are expressed as mean ± SD (*n* = 3). Compared with the control group, # *p* < 0.05. Compared with the dexamethasone group, * *p* < 0.05 and ** *p* < 0.01.

Figure 7 presents the quantitative results of MYHC protein expression in dexamethasone-induced C2C12 cells treated with different-molecular-weight fractions of *H. sinensis* extract. According to Figure 7A, MYHC protein expression significantly decreased after the addition of 25 μM dexamethasone, indicating that MYHC protein was disrupted, confirming the successful induction of muscle atrophy. When different-molecular-weight fractions were added individually, there was no significant change in MYHC protein expression, suggesting that these extracts do not affect MYHC expression under non-atrophy conditions. However, in the dexamethasone-induced atrophy group, only the <3.5 kDa fraction significantly increased MYHC protein expression (*p* < 0.05), indicating its protective effect against dexamethasone-induced muscle damage and its ability to maintain muscle mass. Figure 7B also shows the impact of different-molecular-weight fractions on AMPK protein expression. The results indicate a significant increase in AMPK protein expression in dexamethasone-treated cells (*p* < 0.05), suggesting successful induction of muscle atrophy and activation of AMPK as a compensatory response. The addition of <3.5 kDa and 3.5–10 kDa fractions significantly inhibited AMPK protein expression (*p* < 0.05).

## 4. Discussion

Muscle loss is a condition that naturally occurs with age and involves a reduction in mass, strength, and endurance. This can have a negative impact on quality of life and self-care ability and increase the risk of fractures and falls [20]. Moreover, skeletal muscle plays a vital role in metabolic functions and is closely linked to the risk of metabolic diseases [21]. At present, there are no FDA-approved medications for treating sarcopenia. *H. sinensis*, a medicinal fungus with potential therapeutic effects, is hindered in industrial production by its demanding growth conditions. Recent advancements in fermentation technology present a viable pathway towards commercialization [22]. However, the precise mechanisms of action for the active components from *H. sinensis* have yet to be comprehensively elucidated and remain unreported in the current literature. This study aims to investigate the effects of *H. sinensis* extracts on sarcopenia and to elucidate its mechanism of action through the inhibition of muscle atrophy-related genes.

In this study, extracts of *H. sinensis* were administered to immobilized mice to confirm their effects on muscle endurance and tissue changes. The results revealed a significant improvement in the soleus muscle mass and endurance. The observed enhancements in endurance corroborate and expand upon the findings from prior animal studies investigating fatigue reduction potential. However, there was no notable effect on the gastrocnemius muscle or grip strength. In mammals, skeletal muscles are classified into two types with distinct functions. Type I fibers (slow-twitch fibers) are primarily involved in endurance activities, and type II fibers (fast-twitch fibers) are responsible for rapid movements, but fatigue easily [23]. Thus, the effectiveness of various-molecular-weight fractions of *H. sinensis* extracts was further evaluated using C2C12 cells with dexamethasone damage. Dexamethasone is a glucocorticoid often used for decreasing inflammation symptoms [24]. Its long-term use can lead to muscle atrophy and has been reported on in other studies in an attempt to understand its muscle atrophy mechanisms [25].

This experiment utilized dexamethasone to induce muscle atrophy and activate the AMPK signaling pathway, promoting downstream *Atrogin-1* and *MuRF-1* muscle-specific ubiquitin ligases [26]. The results showed that the water extract fractions with different sizes effectively reduced the expression of *Atrogin-1* and *MuRF-1* genes, which are associated with muscle atrophy. Furthermore, the low-molecular-weight fractions (<3.5 kDa) were also found to enhance the expression of *Myh2* and *Myh7* genes, which play crucial roles in muscle endurance and function [27]. This aligns with current animal experiments demonstrating an increase in soleus muscle mass, which predominantly comprises slow-twitch muscle fibers [28], and is primarily associated with *Myh7* expression. Nonetheless, muscle mass is maintained by a dynamic balance between protein synthesis and degradation [29]. Our study further demonstrates for the first time that the low-molecular-weight fractions (<3.5 kDa) of the *H. sinensis* water extract can significantly increase MYHC protein expression, promoting muscle synthesis by suppressing AMPK protein expression.

Studies of water-soluble polysaccharides from *O. sinensis* have demonstrated various pharmacological effects, including antioxidant, anti-inflammatory, immunomodulatory, and prebiotic properties, showing potential in treating conditions such as diabetes, cancer, intestinal disorders, and atherosclerosis [9,13,30,31]. Alterations in monosaccharide composition, molecular weight, and configuration may be responsible for influencing its pharmacological activity [9]. The anticancer activity of *O. polysaccharides* is correlated with their molecular weight. Those with a molecular weight greater than 16,000 exhibit anticancer properties, including the inhibition of cancer cell metastasis [32]. In contrast, compounds with lower molecular weight show better antioxidant activity [33]. Water-soluble polysaccharide glucogalactomannan (8.1 kDa) isolated from *O. sinensis*, composed of glucose, mannose, and galactose, exhibited significant antioxidant activity [34]. The water-soluble polysaccharide CME-1 (27.6 kDa), isolated from the mycelium, protects RAW264.7 cells from oxidative stress [35]. Studies also indicate that the polysaccharides of *O. sinensis* significantly upregulated metabolic regulators in both exercising and non-exercising skeletal muscles of rats, potentially due to the polysaccharides enhancing endurance and energy metabolism. These antioxidant properties may limit oxidative stress in skeletal muscles during exercise, thereby mitigating related cellular dysfunction and improving exercise endurance [36,37]. A previous study has also indicated that *O. sinensis* mycelium can enhance exercise coordination by increasing muscle endurance and reducing fatigue [38].

For the first time, the current findings propose that the low-molecular-weight fraction (<3.5 kDa) of *H. sinensis* water extract has a protective effect on slow-twitch-type muscle genes. In addition to the previously mentioned genes (*Myh2*, *Myh7*, *MuRF-1*, *Atrogin-1*), we also investigated other genes related to muscle type and growth, including *Myh1*, *MyoD*, and *Myogenin* (Figure S1). The results indicated that the low-molecular-weight fraction (<3.5 kDa) did not have any effect on *Myh1* expression (Figure S1A). Moreover, the expression levels of *MyoD* and *Myogenin* showed an upward trend (Figure S1B,C). *MyoD* plays a crucial role in muscle cell differentiation and regeneration, while *Myogenin* is a key gene involved in the muscle differentiation process [39]. Additionally, we observed the morphology of C2C12 cells through H&E staining (Figures S2 and S3). The results showed that after treatment with the low-molecular-weight fraction (<3.5 kDa), the cell diameter was significantly larger compared to the control group, and it effectively promoted muscle cell differentiation, contributing to muscle development. These findings demonstrate that *H. sinensis* extract exhibits a notable capacity to enhance muscle cell differentiation and maintain cellular functionality through the upregulation of muscle-related genes. This distinct mechanistic pathway, which represents a new finding compared to previous investigations, indicates that the low-molecular-weight fraction has considerable potential for preserving muscle integrity and supporting overall muscle health during endurance-based physical activities.

As people age, they experience increased protein loss, which can lead to muscle loss. This is often worsened by decreased appetite and metabolic disorders in older adults [40]. The aqueous extraction methodology of *H. sinensis* demonstrates enhanced antioxidant properties [41], with water-soluble polysaccharides potentially serving as the primary mechanism in mitigating muscle atrophy. This finding aligns with our current gene expression analysis, specifically regarding the suppression of atrophy-associated genes (*MuRF-1* and *Atrogin-1*) in the >10 kDa + Dex group. Consequently, integrating the findings from this investigation with established resistance training protocols and optimal protein intake presents a promising approach for enhancing protein synthesis while reducing degradation, thus promoting the maintenance of muscle mass during healthy aging [42].

## 5. Conclusions

This study has shown that *H. sinensis* extract has the potential to slow down sarcopenia by preserving slow-twitch muscle fibers, thus reducing muscle atrophy caused by steroid treatments, diseases, or aging. This can improve muscle endurance in older adults and overall muscle function. This research presents new potential applications for *H. sinensis* extracts in preventing sarcopenia and maintaining muscle health. Further studies are needed to explore its mechanisms and clinical application value.

## Data Availability

The data presented in this study are available on request from the corresponding author.

## Supplementary Materials

The following are available online at https://www.mdpi.com/article/10.3390/cimb46120839/s1: Figure S1: The effect of the low-molecular-weight fraction (<3.5 kDa) of *H. sinensis* water extract on other genes related to muscle growth or atrophy. Figure S2: The effect of the low-molecular-weight fraction (<3.5 kDa) of *H. sinensis* water extract on C2C12 myotube cell diameter. Figure S3: Promotion of C2C12 myotube differentiation by the low-molecular-weight fraction (<3.5 kDa) of *H. sinensis* water extract as observed in H&E-stained images.
