# Supplementary material for: Prevention of Muscle Atrophy by Low-Molecular-Weight Fraction from Hirsutella sinensis Mycelium
Source: Curr Issues Mol Biol. 2024 Dec 12;46(12):14033–44. doi: 10.3390/cimb46120839 (PMC11727312; doi:10.3390/cimb46120839)
Supplement: Supplementary file 1 [file cimb-46-00839-s001.zip › cimb-3279682-supplementary.pdf]

Supporting Information

Prevention of Muscle Atrophy by Low-Molecular-Weight Fraction from *Hirsutella sinensis* Mycelium

Yi-Wen Chen [1,†], Tsung-Ju Li [1,†], Li-Ching Wang [2], Bi-Hua Yang [1], Yen-Lien Chen [1], Chin-Chu Chen [1,3,4,5,*] and Hsin-Tang Lin [2,*]

1 Biotech Research Institute, Grape King Bio Ltd., Taoyuan City 325, Taiwan; yiwen.chen@grapeking.com.tw (Y.-W.C.); tsungju.li@grapeking.com.tw (T.-J.L.); sybil.yang@grapeking.com.tw (B.-H.Y.); Ian.chen@grapeking.com.tw (Y.-L.C.)
2 Department of Food Safety, National Chung Hsing University, Taichung City 402, Taiwan; gash94272abc@gmail.com
3 Department of Food Science, Nutrition, and Nutraceutical Biotechnology, Shih Chien University, Taipei City 104, Taiwan
4 Institute of Food Science and Technology, National Taiwan University, Taipei City 106, Taiwan
5 Department of Bioscience Technology, Chung Yuan Christian University, Taoyuan City 320, Taiwan
* Correspondence: gkbioeng@grapeking.com.tw (C.-C.C.); linhs@nchu.edu.tw (H.-T.L.); Tel.: +886-04-2284-0867 (ext. 203213) (H.-T.L.)

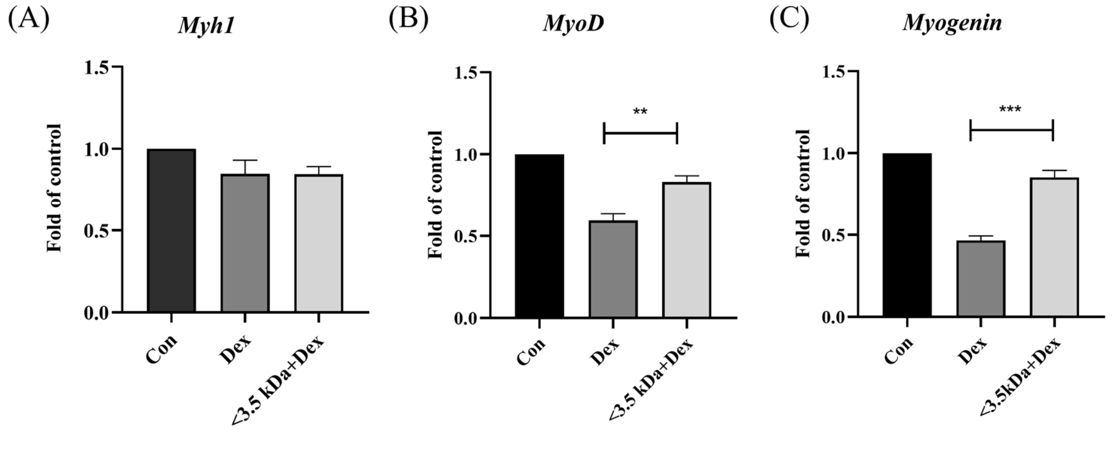

Figure S1. The effect of low molecular weight fraction (<3.5 kDa) of *H. sinensis* water extract on other genes related to muscle growth or atrophy. (A) *Myh1* (B)*MyoD* (C) *Myogenin.* Compared with the Dex group **p<0.01,***p < 0.001.

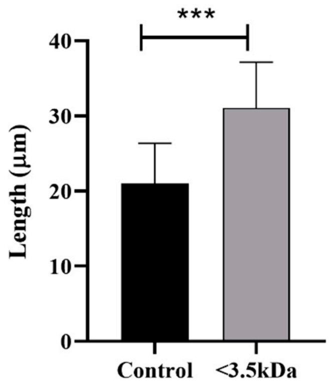

Figure S2. The effect of low molecular weight fraction (<3.5 kDa) of *H. sinensis* water extract on C2C12 myotube cell diameter. Results were expressed as mean ± SD (n = 50). Compared with the control group ***$p <$ 0.001.

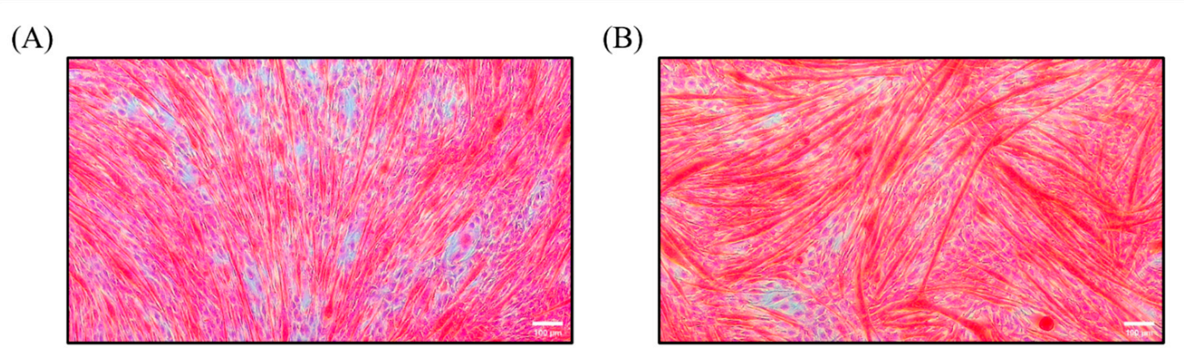

Figure S3. Promotion of C2C12 myotube differentiation by the low-molecular-weight fraction (<3.5 kDa) of *H. sinensis* water extract as observed in H&E-stained images. (A) Control group, (B) <3.5kDa.
